# Evaluating the Prevalence and predictors of anxiety and depression among elementary and high school educators in Canada.

**DOI:** 10.1192/j.eurpsy.2025.671

**Published:** 2025-08-26

**Authors:** A. Belinda, Y. O. Wei, R. D. L. D. Dias, A. Orimalade, P. Brett-MacLean, V. I. O. Agyapong

**Affiliations:** 1 University of Alberta, Edmonton; 2 Dalhousie University, Halifax, Canada

## Abstract

**Introduction:**

Globally, anxiety and depression are primary contributors to work disability and are associated with the mental and physical well-being of educators.Anxiety and depressive disorders result in poor mental health, great human misery, enormous loss in economic output and increased public health and economic burden.

**Objectives:**

To determine the prevalence and independent predictors of likely Generalized Anxiety Disorder (GAD) and likely Major Depressive Disorder (MDD) among teachers in Newfoundland and Labrador, Alberta, and Nova Scotia.

**Methods:**

The study utilized a cross-sectional design. Educators in the three Canadian provinces completed an online survey after enrolling on the Wellness4Teachers program, a daily supportive text messaging program. Likely GAD and likely MDD among subscribers were respectively assessed using the Generalized Anxiety Disorder-7 scale and Patient Health Questionnaire-9. Data was analyzed with SPSS version 28.

**Results:**

Overall,763 out of the 1912 subscribers of the Wellness4Teachers program completed the survey, resulting in a 39.91% response rate. The prevalence of likely MDD was 55.7%, and likely GAD was 46.0%. Participants who experience high stress were 7.24 times more likely to experience MDD (OR = 7.24; 95% CI: 4.22–12.42) and 7.40 times more likely to experience GAD (OR = 7.40; 95% CI: 4.63–11.80) than those with mild to moderate stress. Again, participants with emotional exhaustion were 4.92 times more likely to experience MDD (OR = 4.92; 95% CI: 3.01–8.05) and 4.34 times more likely to experience GAD (OR = 4.34; 95% CI: 2.47–7.62) than those who did not. Similarly, respondents with low resilience were 3.01 times more likely to experience likely GAD compared to those with normal to high resilience (OR =3.01; 95% CI: 2.03-7.62). Sociodemographic and work-related variables did not independently predict the presence of likely GAD and likely MDD.

**Conclusions:**

The current study reinforces the need for governments and policymakers in the education sector to implement appropriate and comprehensive mental health support programs to address the unique stressors faced by educators, reduce emotional exhaustion and improve resilience as a way to reduce anxiety and depression, promote their well-being and enhance the quality of educational delivery.

**Disclosure of Interest:**

None Declared

